# A new species of *Isospora* (Apicomplexa: Eimeriidae) from the yellow warbler, *Setophaga petechia* (L.) (Passeriformes: Parulidae: Parulinae), in Oklahoma, USA

**DOI:** 10.1016/j.crpvbd.2022.100096

**Published:** 2022-07-18

**Authors:** Chris T. McAllister, John A. Hnida

**Affiliations:** a Science and Mathematics Division, Eastern Oklahoma State College, Idabel, OK 74745, USA; b Department of Microbiology and Immunology, Midwestern University, Glendale, AZ, 85308, USA

**Keywords:** *Setophaga petechia*, Passeriformes, Parulidae, Apicomplexa, *Isospora*, New species

## Abstract

*Isospora fitzpatricki* n. sp. is described from a yellow warbler, *Setophaga petechia* (L.), from McCurtain County, Oklahoma, USA. Oöcysts of the new species are subspheroidal to ovoidal with a smooth bi-layered wall, measure (L × W) 24.2 × 20.4 μm, and have a length/width (L/W) ratio of 1.2; a micropyle and oöcyst residuum are both absent, but polar granule(s) are present. Sporocysts are ovoidal to ellipsoidal and measure 14.5 × 9.2 μm, L/W ratio of 1.6; a knob-like Stieda body is present as well as a distinct rounded sub-Stieda body. The sporocyst residuum is composed of various-sized granules in a compact rounded or irregular mass or dispersed between and across the sporozoite. The oöcysts of *I. fitzpatricki* n. sp. can be differentiated from five other congeners reported from members of the New World warbler family Parulidae from either Mexico, Costa Rica, or Brazil, by being larger on average as well as by possessing a prominent rounded sub-Stieda body, sporozoite striations, and an posterior refractile body. Information is also presented on an *Isospora* sp. being passed by *S. petechia* that we choose not to describe as a putative novel species of *Isospora* at this time. These two isosporans represent the first coccidians reported from *S. petechia* and, more importantly, the first known from the members of the Parulidae in the USA.

## Highlights

• A description of a new species of *Isospora* is provided from a yellow warbler, *Setophaga petechia.*
• This is the first report of a coccidian described from any bird in the warbler family Parulidae from the USA.
• Metrical and morphological information is provided for *Isospora* sp. from *S. petechia.*

## Graphical abstract

**Figure:**
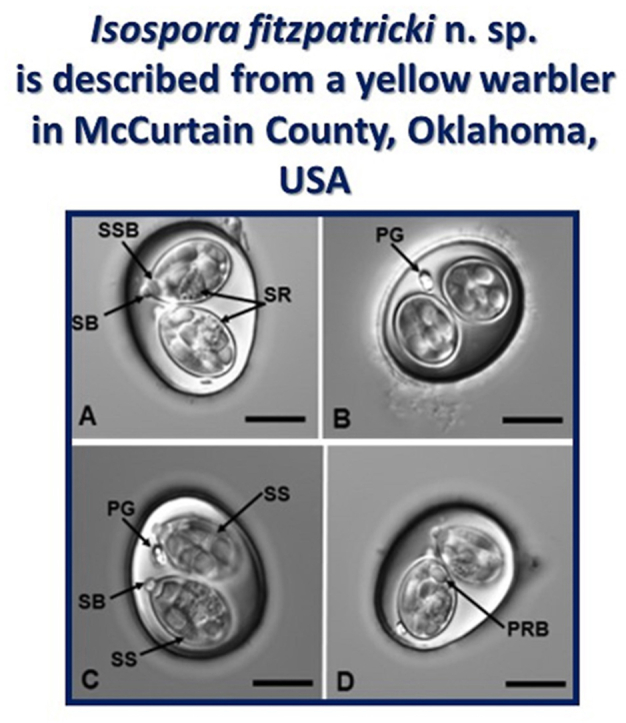

## 1 Introduction

The yellow warbler, *Setophaga petechia* (L.) (Passeriformes: Parulidae: Parulinae) is a long-distance migrant that ranges from northern Alaska and northern Canada to Labrador south through the central plateau of Mexico to Panama and central Peru, northern Bolivia, and Amazonian Brazil, and east through the West Indies (Pashley, 1988a, b; Pashley & Hamilton, 1990). The species breeds in most of North America from Alaska and far northern Canada south to northwest Mexico and winters from Mexico to Peru (Cornell Lab of Ornithology, 2019). In Oklahoma, it is widespread in the state but is more common in the eastern portion (Tekiela, 2002; Kuhnert, 2004). It inhabits a variety of habitat including open scrub, thickets, second-growth woodland, farmland, and gardens, riparian woodlands, swamps, and along other watercourses. This bird is a copious feeder of insects, especially caterpillars and will occasionally take small fruits/berries or probes in flowers (Lack, 1976; Tekiela, 2002).

Phylogenetic analyses of sequences of nuclear and mitochondrial DNA (Lovette et al., 2010) indicated that all species formerly placed in the genus *Dendroica* G.R. Gray, one species formerly placed in *Wilsonia* Bonaparte (*W*. *citrina* Boddaert), and two species formerly placed in *Parula* Bonaparte (*P*. *americana* (L.) and *P*. *pitiayumi* (Vieillot)) form a clade with the single species customarily placed in the genus *Setophaga* Swainson (*S*. *ruticilla* (L.)). Thus, the generic name *Setophaga* has priority for this clade (Chesser et al., 2011). There are 34 recognized subspecies of *S. petechia* (Gill et al., 2022).

The New World warbler family Parulidae includes 111 species of New World or wood-warblers (Winkler et al., 2020). To date, only five species (4.5%) have been reported to harbor isosporan coccidians. All of these coccidians have been previously described from birds collected in Mexico, Costa Rica, or Brazil (Berto et al., 2010, Berto et al., 2014; Keeler et al., 2014; Salgado-Miranda et al., 2016; Mello et al., 2022). Nothing, however, is known of the coccidian parasites of *S. petechia*. Here, we describe a new species of *Isospora* as well as document another *Isospora* sp. from *S. petechia* from Oklahoma, USA.

## 2 Materials and methods

### 2.1 Sample collection

Faecal samples were collected during March 2022 from the rectum of two adult *S. petechia* found dead in McCurtain County, Oklahoma, USA, and examined for coccidian parasites. Briefly, a mid-ventral incision was made to expose the gastrointestinal tract; faeces were taken from the rectum of each bird. Samples were placed in individual vials containing 2.5% (K_2_Cr_2_O_7_) aqueous potassium dichromate at 1:6 (v/v).

### 2.2 Morphological analyses

Samples were further examined for coccidia *via* flotation in 15-ml conical centrifuge tubes (with centrifugation) containing Sheather’s sugar solution (specific gravity: 1.30) using an Olympus BX43 light microscope (Olympus Corporation, Center Valley, Pennsylvania, USA). A positive faecal sample was placed in a Petri dish containing a small layer of K_2_Cr_2_O_7_ for 48–72 h to allow sporulation. All morphological measurements are reported in micrometres (μm) and are given as the range followed by the mean in parentheses. Photographs were taken using Nomarski interference-contrast optics at ×1000 magnification. Oöcysts were *c*.45 days-old when measured and photographed. Descriptions of oöcysts and sporocysts follow the standard guidelines of Wilber et al. (1998) including oöcyst length (L) and width (W), their ranges and ratios (L/W), micropyle (M), oöcyst residuum (OR), polar granule(s) (PG), sporocyst length (L) and width (W), their ranges and ratio (L/W), sporocyst (SP), Stieda body (SB), sub-Stieda body (SSB), para-Stieda body (PSB), sporocyst residuum (SR), sporozoites (SZ) anterior (ARB) and posterior (PRB) refractile bodies, and nucleus (N).

## 3 Results

One of two (50%) yellow warblers were found to be passing oöcysts of a new species of *Isospora* as well as an *Isospora* sp., the former of which is described herein as new. This material is described below.

### 3.1 *Isospora fitzpatricki* n. sp.

#### 3.1.1 Taxonomic summary

*Type-host*: *Setophaga petechia* (L.) (Passeriformes: Parulidae), yellow warbler, adult male, collected 3 March 2022.

*Type-locality*: Hochatown off US 259 (34°10′17.0286″N, −94°45′05.7414″W), McCurtain County, Oklahoma, USA.

*Type-material*: Photosyntypes of sporulated oöcysts are deposited as HWML 216810.

*Site in host*: Unknown; oöcysts passed in faeces.

*Prevalence*: 1 of 2 (50%) birds examined.

*ZooBank registration*: To comply with the regulations set out in Article 8.5 of the amended 2012 version of the International Code of Zoological Nomenclature (ICZN, 2012) details of the new species have been submitted to ZooBank. The Life Science Identifier (LSID) of the article is urn:lsid:zoobank.org:pub:57C64E12-4CC1-4D34-BFEB-B9C42F8AB045. The LSID of the new name *Isospora fitzpatricki* is urn:lsid:zoobank.org:act:5BA074E0-B31F-44A7-861F–37F97FB0F125.

*Etymology*: The specific epithet is in honor of Lloyd Charles Fitzpatrick, Emeritus Professor of Biology, University of North Texas, Denton, Texas, USA; he served as major professor for CTM’s doctoral degree.

#### 3.1.2 Description

**Fig. 1:**
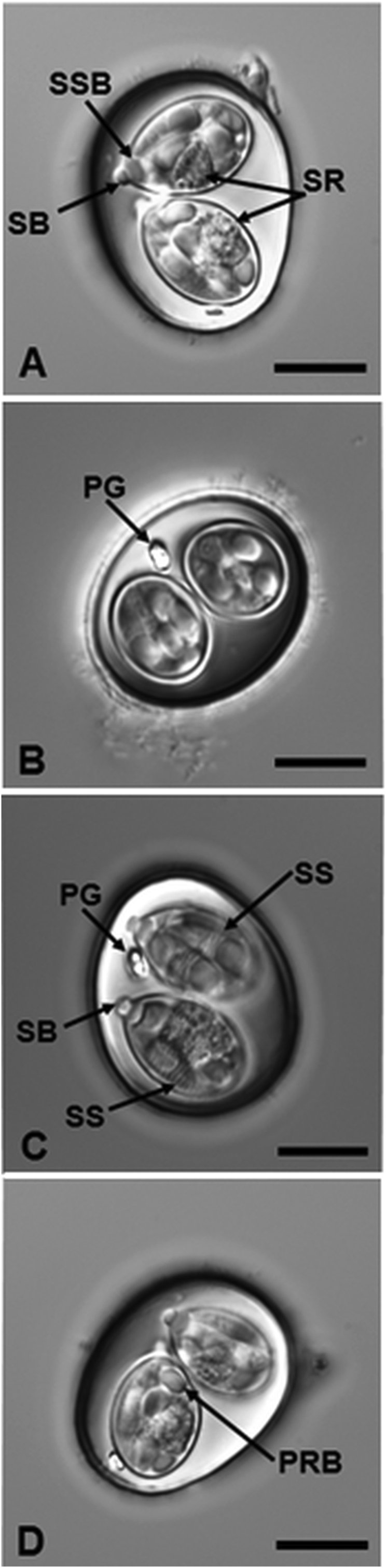
Nomarski interference-contrast photomicrographs of sporulated oöcysts of *Isospora fitzpatricki* n. sp. from the yellow warbler *Setophaga petechia*. *Abbreviations*: polar granule (PG); posterior refractile body (PRB); Stieda body (SB); sub-Stieda body (SSB); sporocyst residuum (SR); sporozoite striations (SS). *Scale-bars*: 10 μm.

**Fig. 2:**
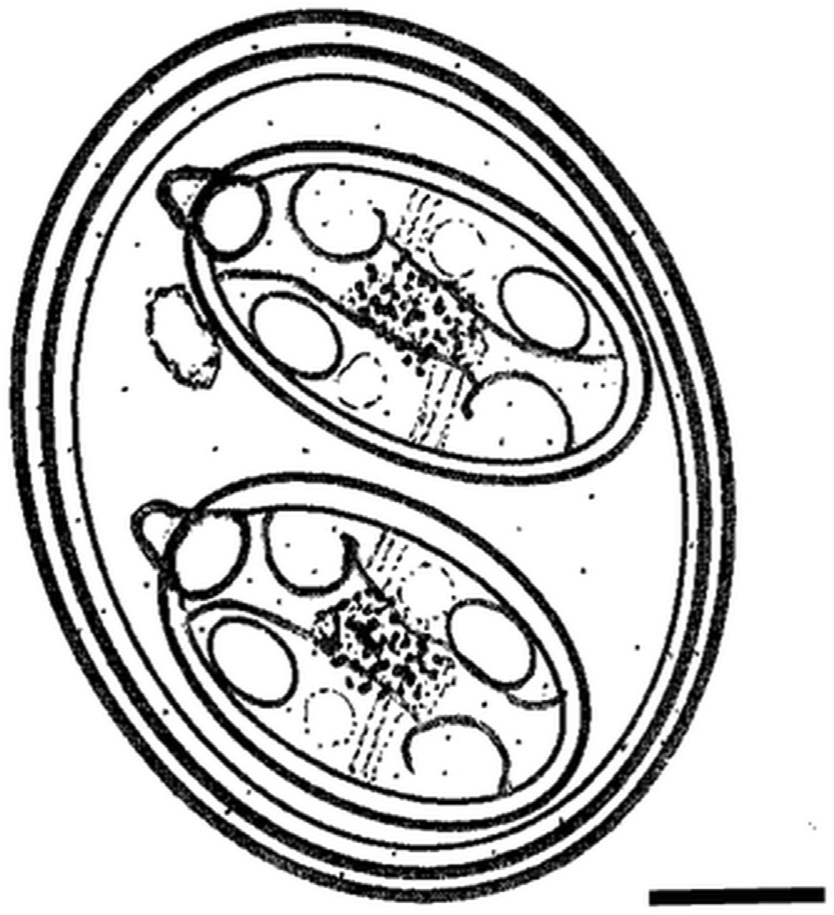
Composite line drawing of *Isospora fitzpatricki* n. sp. *Scale bar*: 5 μm.

[Based on 18 oöcysts and 18 sporocysts; Fig. 1, Fig. 2] Oöcyst subspheroidal to ovoidal, 21–29 × 18–24 (24.2 × 20.4); L/W ratio: 1.1–1.3 (1.20). Wall bi-layered, 1.0–1.5 (*c*.1.2) thick; outer layer smooth (*c*.0.8), colorless to light tan; inner layer (*c*.0.4) darker. Micropyle and oöcyst residuum both absent, but one (typically) to 2 polar granules present.

Sporocysts 2, ovoidal to ellipsoidal, 13–18 × 8–10 (14.5 × 9.2); L/W ratio: 1.4–1.8 (1.6). Stieda body knob-like, 1.0–1.2 (1.1) high × 1.3–1.9 (1.5) wide; sub-Stieda body prominent, rounded, 1.0–1.7 (1.3) high × 1.7–2.5 (2.1) wide; para-Stieda body absent. Sporocyst residuum composed of various-sized granules in compact rounded, irregular mass, or dispersed between and across sporozoites.

Sporosoites 4, vermiform (not measured) with striations at anterior end, centrally located nucleus and ellipsoidal posterior refractile bodies; anterior refractile bodies absent.

#### 3.1.3 Remarks

Five species of *Isospora* have been previously described from parulid birds (Berto et al., 2010, 2014; Keeler et al., 2014; Salgado-Miranda et al., 2016; Mello et al., 2022) (Table 1). None, however, have been reported from any member of the family in the USA. When the new species is compared to these isosporans, it is most similar to three of them, including *Isospora basileuterusi* Mello & Berto, 2022 from the golden-crowned warbler *Basileuterus culicivorus* (Deppe) from Brazil, *Isospora piachobrai* Berto, Flausino, Luz, Ferreira & Lopes, 2010 from the masked yellowthroat, *Geothlypis aequinoctialis* (Gmelin) from Brazil, and *Isospora orbisrenitas* Keeler, Yabsley, Adams & Hernandez, 2014 from the rufous-capped warbler, *Basileuterus rufifrons* (Swainson) from Costa Rica. The new species differs from these as follows: (i) oöcysts of *Isospora fitzpatricki* n. sp. are larger (on average) than each of them (see Table 1); (ii) although the knob-like Stieda body is similar among all of them, when compared to the new species, the sub-Stieda body of all three previously described congeners is large and trapezoidal *versus* the prominent rounded sub-Stieda body of *I. fitzpatricki* n. sp.; (iii) sporozoites of the new species have striations (see Fig. 1C) and no striations were mentioned in the species description or delineated on the line drawing for the sporozoites of *I. basileuterusi*; and (iv) sporozoites of *I. basileuterusi* are described having both an anterior refractile body (ARB) and posterior refractile body (PRB), whereas those of *I. fitzpatricki* n. sp. possess only the PRB. Based on these notable metrical and morphological differences, we believe that *I. fitzpatricki* is a genuine new species.

**Table 1:** Comparison of the sporulated oöcysts of *Isospora* spp. from members of the family Parulidae

Species	Type-host^a^ (Type-locality)	Oöcyst shape, size, features	Sporocyst shape, size, features	Reference
*I. basileuterusi* Mello & Berto, 2022	*Basileuterus culicivorus* (Deppe) (Brazil)	Ellipsoidal to ovoidal; 25.2 × 21.1; L/W: 1.2; 22–28 × 17–23; M, OR: both ‒; PG: + 1 (1–3)	Ellipsoidal to lemon-shaped; 15.3 × 9.5; L/W: 1.6; 14–17 × 8–11; SB (K), SSB (T), SR: all +	Mello et al. (2022)
*I. cardellinae* Salgado-Miranda, Zepeda-Velazquez, Garcia-Conejo, Galindo-Sanchez, Janczur & Soriano-Vargas, 2016	*Cardellina rubra* (Swainson) (Mexico)	Subspheroidal; 26.6 × 25.4; L/W: 1.1; 23–28 × 23–27; M, OR: both ‒; PG: –	Ovoidal; 19.0 × 12.0; L/W: 1.7; 18–20 × 11–13; SB (K), SSB (T), SR: all +	Salgado-Miranda et al. (2016)
*I. celata* Berto, Medina, Zepeda-Velázquez, García-Conejo, Galindo-Sánchez, Janczur & Soriano-Vargas, 2016	*Leiothlypis celata* (Say) (Mexico)	Subspheroidal; 28.0 × 26.0; L/W: 1.1; 27–30 × 25–28; M, OR: both –; PG: –	Ovoidal; 18.0 × 13.0; L/W: 1.4; 15–20 × 11–14; SB (K), SSB (I), SR: all +	Berto et al. (2014)
*I. fitzpatricki* n. sp.	*Setophaga petechia* (L.) (Oklahoma, USA)	Subspheroidal to ovoidal; 24.2 × 20.4; L/W: 1.2; 21–29 × 18–24; M, OR: both –; PG: 1 (1–2)	Ovoidal; 14.5 × 9.2; L/W: 1.6; 13–18 × 8–10; SB (K), SSB (R), SR: all +	Present study
*I. orbisreinitas* Keeler, Yabsley, Adams & Hernandez, 2014	*Basileuterus rufifrons* (Swainson) (Costa Rica)	Subspheroidal to ovoidal; 24.3 × 22.3; L/W: 1.0; 21–28 × 19–25; M, OR: both ‒; PG: +/− (0–4)	Ovoidal; 16.0 × 11.8; L/W: 1.4; 12–19 × 10–14; SB (K), SSB (T), SR: all +	Keeler et al. (2014)
*I. piacobrai* Berto, Flausino, Luz, Ferreira & Lopes, 2010	*Geothlypis aequinoctialis* (Gmelin) (Brazil)	Subspheroidal to ovoidal; 23.5 × 21.6; L/W: 1.1; 21–26 × 20–24; M, OR: both ‒; PG: + (1)	Ovoidal; 15.8 × 10.5; L/W: 1.5; 15–17 × 9–12; SB (K), SSB (T), SR: all +	Berto et al. (2010)
*Isospora* sp.	*Setophaga petechia* (L.) (Oklahoma, USA)	Subspheroidal to spheroidal; 27.0 × 25.2; L/W: 1.1; 25–28 × 23–27; M, OR: both ‒; PG: + (1)	Ovoidal; 15.4 × 11.8; L/W: 1.3; 15–16 × 11–12; SB (K), SSB (T), SR: all +	Present study

Note: Measurements are in micrometres (μm) and abbreviations follow Wilber et al. (1998).

Abbreviations: K, knob-like; T, trapezoidal; I, irregular, barely discernible; R, round; +, present; –, absent.

a Current name.

### 3.2 *Isospora* sp.

#### 3.2.1 Taxonomic summary

*Host*: *Setophaga petechia* (L.) (Passeriformes: Parulidae), yellow warbler, adult male, collected 3 March 2022.

*Locality*: Hochatown off US 259 (34°10′17.0286″N, −94°45′05.7414″W), McCurtain County, Oklahoma, USA.

*Voucher material*: Photovoucher of sporulated oöcyst is deposited as HWML 216811.

*Site in host*: Unknown; oöcysts passed in faeces.

*Prevalence*: 1 of 2 (50%) birds examined.

#### 3.2.2 Description

**Fig. 3:**
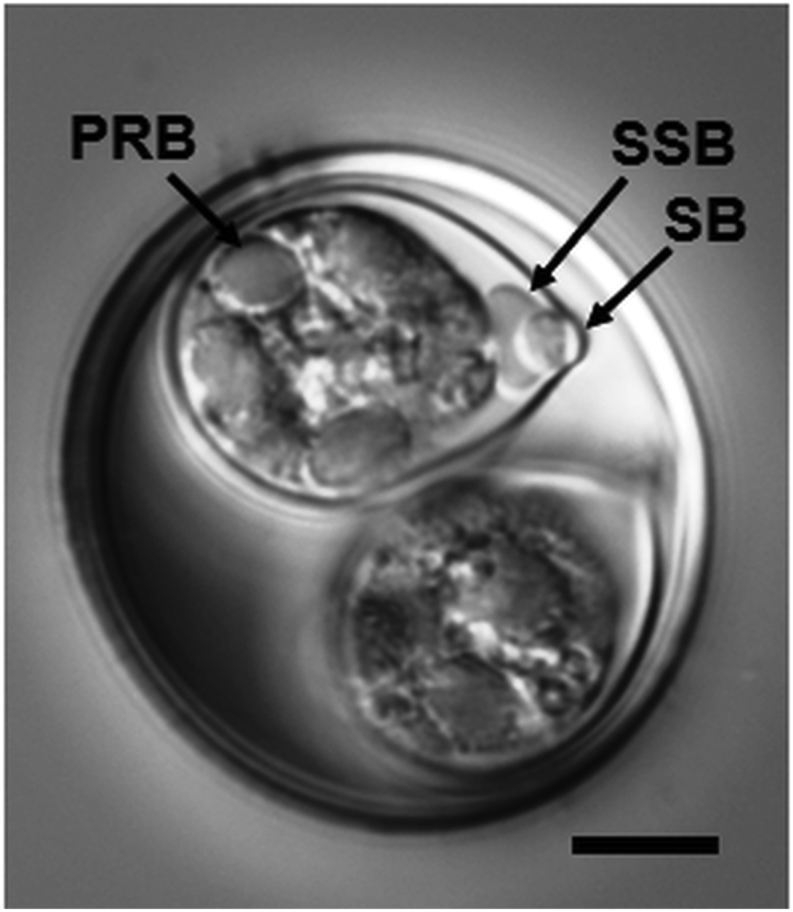
Nomarski interference-contrast photomicrograph of sporulated oöcyst of *Isospora* sp. from the yellow warbler *Setophaga petechia*. *Abbreviations*: posterior refractile body (PRB); Stieda body (SB); sub-Stieda body (SSB). *Scale-bar*: 5 μm.

[Based on 5 oöcysts and 5 sporocysts; Fig. 3.] Oöcyst subspheroidal to ovoidal, 25–28 × 23–27 (27.0 × 25.2); L/W ratio 1.0–1.1 (1.10). Wall bi-layered, 1.0–1.5 (*c*.1.2) thick; outer layer (*c*.0.8), smooth and colorless to light tan; inner layer (*c*.0.4) darker. Micropyle and oöcyst residuum both absent, one polar granule present.

Sporocysts 2, ovoidal, 15–16 × 11–12 (15.4 × 11.8); L/W ratio 1.3–1.4 (1.3). Stieda body knob-like, 0.5–0.8 (0.6) high × 1.3–1.8 (1.6) wide. Sub-Stieda body compartmentalized; outer portion trapezoidal, 2.2–3.0 (2.6) high × 3.8–4.7 (4.1) wide; central portion rounded, 1.2–1.7 (1.5) high × 1.6–2.1 (1.9) wide. Para-Stieda body absent. Sporocyst residuum as large, diffuse mass of various-sized granules located between and obscuring sporozoites.

Sporozoites 4, difficult to observe, with ellipsoidal posterior refractile bodies.

#### 3.2.3 Remarks

Unfortunately, only five oöcysts were recovered from this faecal sample. This form does, however, represent a species of *Isospora* possessing one of the largest oöcysts (on average) of any parulid coccidian, except for *Isospora celata* Berto, Medina, Zepeda-Velázquez, García-Conejo, Galindo-Sánchez, Janczur & Soriano-Vargas, 2016 (see Table 1). Nevertheless, *I. celata* does not possess a polar granule and its sub-Stieda body is barely discernible which differs considerably from the *Isospora* sp. described here. In addition, the oöcysts of *Isospora orbisreinitas* (Table 1) are equivalent in the range of measurements as well as the possession of a compartmentalized sub-Stieda to *Isospora* sp. However, because few oöcysts were found, we choose to take the conservative approach and, until additional samples are available, we decline to name this species at this time.

## 4 Discussion

Yellow warblers are one of the most numerous warblers in North America but their populations have been slowly declining, and have decreased by 25% between 1966 and 2014, according to the North American Breeding Bird Survey (Sauer et al., 2014). Populations are jeopardized in some areas by loss of riparian habitat in combination with heavy brood parasitism by brown-headed cowbirds, *Molothrus ater* (Boddaert), and shiny cowbirds, *Molothrus bonariensis* (Gmelin) (Ehrlich et al., 1988, Ehrlich et al., 1992, Weatherhead, 1989)

## 5 Conclusion

Our comparison of oöcysts of *I. fitzpatricki* n. sp. with oöcysts of *Isospora* spp. previously described from other Western Hemisphere parulid birds supports our designation of a novel species. The new species is the first known from members of its family in the USA. As such, we consider *I. fitzpatricki* new to science, which, to date, becomes the sixth species of *Isospora* described from a host in this family of passeriform birds. We also document an unidentified species of *Isospora* from this host that requires additional samples. Additional parulid birds should be examined for coccidia, particularly those that occur in North America.

## Funding

Official funding for this study was not available.

## Ethical approval

Ethical Approval for collecting was granted per the 10.13039/100014728Oklahoma Department of Wildlife Conservation Scientific Collecting Permit No. 1551646 to CTM. All institutional, national, and international guidelines for the care and use of animals were followed.

## CRediT author statement

This study was designed by both authors. Field collections were performed by CTM. Laboratory procedures for recovery of oöcysts were performed by CTM. Laboratory procedures for measurements, photomicrographs, and isolation of oöcysts were performed by JAH. CTM drew the coccidian oöcyst. The manuscript was written by both authors and subsequently revised by both authors. Both authors read and approved the final manuscript.

## Data availability

Photosyntypes and a photovoucher are accessioned into the Harold W. Manter Laboratory of Parasitology (HWML), University of Nebraska, Lincoln, Nebraska, USA. The host voucher was accessioned into the Eastern Oklahoma State College (EOSC) Collection, Idabel, Oklahoma, USA.

## Declaration of competing interests

The authors declare that they have no known competing financial interests or personal relationships that could have appeared to influence the work reported in this paper.
